# An Unusual Case of Mammary Paget's Disease Diagnosed Using Dynamic Contrast-Enhanced MRI

**DOI:** 10.1155/2013/206235

**Published:** 2013-03-30

**Authors:** Eleonora Gaspari, Aurora Ricci, Valeria Liberto, Angela Lia Scarano, Maria Fornari, Giovanni Simonetti

**Affiliations:** Department of Diagnostic Imaging, Molecular Imaging, Interventional Radiology and Radiation Therapy, University Hospital Tor Vergata, Viale Oxford 81, 00133 Rome, Italy

## Abstract

Mammary Paget's disease is a rare presentation of breast cancer. At clinical examination, it is characterized by skin lesions of the nipple-areola complex, almost always a sign of malignancy. In fact, it is often associated with an underlying mammary ductal carcinoma in situ (DCIS) or invasive carcinoma. An underlying carcinoma is also common in women with negative mammography and ultrasound (US); in these cases, magnetic resonance imaging (MRI) is a diagnostic tool useful in the detection of occult cancer. We described an unusual case of mammary Paget's disease with underlying DCIS, in a patient without nipple-areola complex alterations and/or palpable lump. On suspicion of Paget's disease, the patient underwent MRI examination that proved useful for an accurate diagnosis. Biopsy confirmed dynamic MRI findings.

## 1. Introduction

Paget's disease of the breast is an uncommon disease, accounting for 1%–4.3% of all breast tumors, often associated with underlying ductal carcinoma in situ (DCIS) [1, 2]. The diagnosis of Paget's disease is generally made on the basis of clinical findings confirmed by biopsy. The patients often present itching, erythema, scaly skin, bloody nipple discharge, and nipple erosion or ulceration. Paget's disease clinically occults in approximately 10%–28% of cases identified by histopathological evaluation after mastectomy for in situ or invasive breast carcinoma [3]. Mammographic and ultrasonographic findings are not specific for malignancy. Magnetic resonance imaging (MRI) is a diagnostic tool useful in the detection of clinically occult cancer, with unspecific signs at mammographic and ultrasonographic examinations. Furthermore MRI has an important role in the preoperative planning to establish conservative or demolitive surgical treatment.

We describe an unusual case of Paget's disease arrived to our observation for a routinely control. The patient had still no traditional cutaneous alteration of Paget's disease.

## 2. A Case Presentation

**Figure 1:**
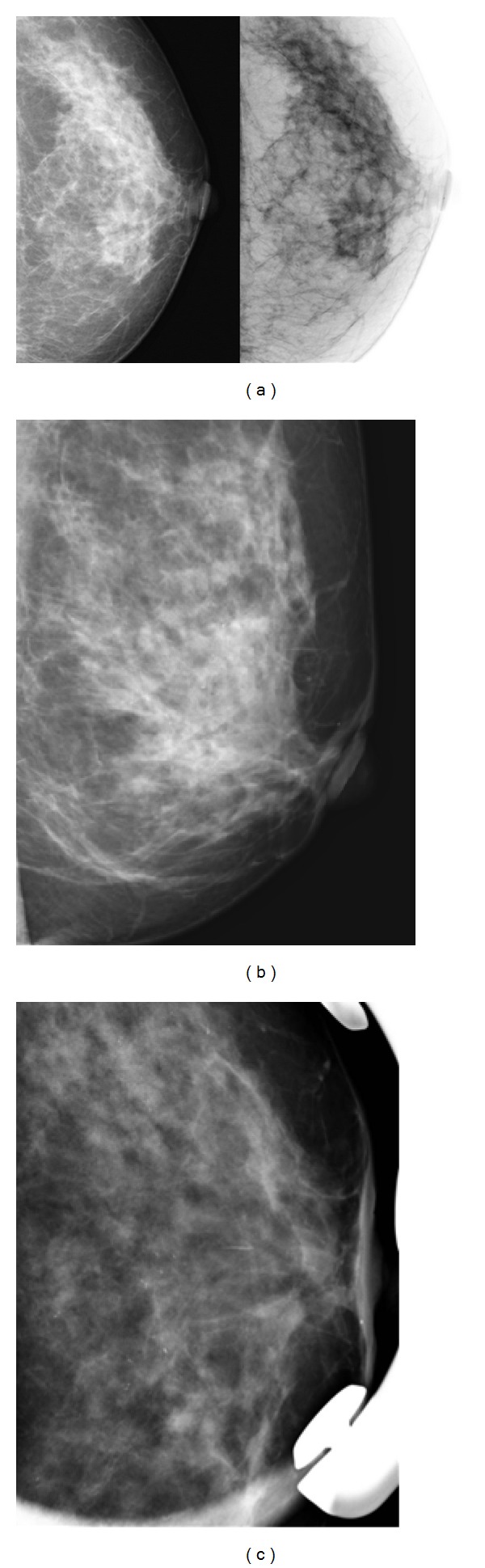
Left craniocaudal projection (a), medio-lateral oblique projection (b), and magnification view (c) show numerous and inhomogeneous microcalcifications in the subareolar region, not associated to underlying mass, architectural distortion, and nipple retraction.

**Figure 2:**
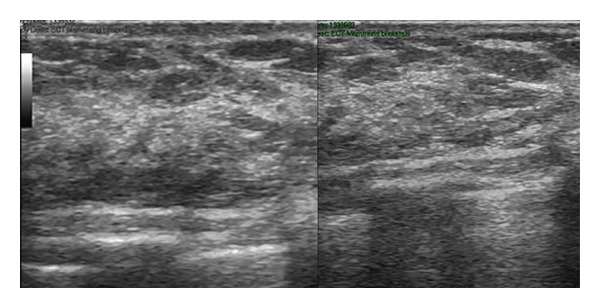
US does not show significant alterations in the subareolar region of the left breast.

**Figure 3:**
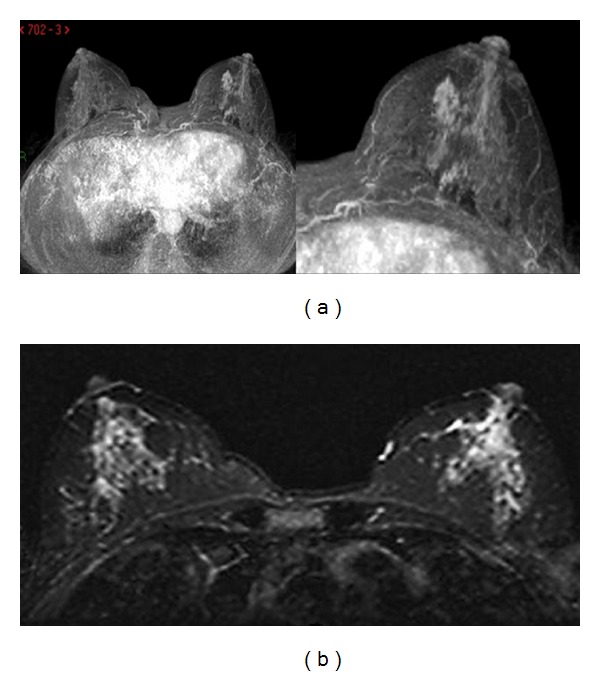
T1 weighted high-resolution isotropic volume examination (THRIVE) (a) and T2 weighted short tau inversion recovery (STIR) sequences (b) show contrast enhancement of the left nipple extending to subareolar region.

**Figure 4:**
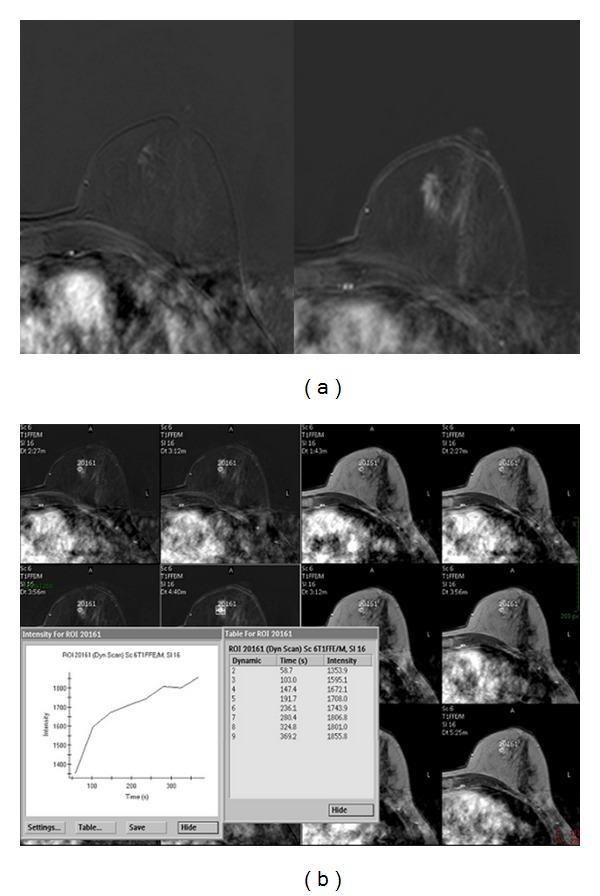
(a) Early and late dynamic contrast-enhanced sequences show a slow and progressive contrast enhancement of the left nipple extending to subareolar region. (b) The time-signal intensity curve appears slow and progressive.

A 56-year-old woman was referred to our institution for breast cancer annual screening. A preliminary clinical examination of the breasts did not show alterations of nipple-areola complex. The patient reported just the presence of itchiness to the left nipple. Physical examination of the breast did not detect the presence of palpable mass, and axillary lymphadenopathy was absent. Bilaterally, nipple discharge was not present. Personal and familiar anamneses were negative for breast cancer. The patient was postmenopausal for 5 years and did not have a hormone replacement therapy. Previous annual mammograms were normal. A digital mammography, performed with GE Senographe DS (General Electric, Milwaukee, USA), using standard projections, did not reveal asymmetric radiopacities or nodular areas bilaterally (Figure 1). It identified, in the subareola region of the left breast, numerous heterogeneous (linear, partially branched, and fine powdery appearance) microcalcifications distributed in a small area, not corresponding to a clearly configured addensative area. US was unremarkable bilaterally (Figure 2). Microcalcifications described were absent in previous controls and did not appear suspicious for malignancy. Although the best decision would be to perform subsequent 4–6 months mammograms and a close followup to evaluate the area of microcalcifications, the patient underwent MRI examination in consideration of the symptomatology. Dynamic contrast-enhanced MRI was performed with a 1.5 T unit (Gyroscan Intera, Philips Medical Systems, Best, The Netherlands) equipped with 4 channels reception dedicated coil. MRI images were acquired on axial planes with FFE-T1 and TSE-T2 weighted sequences, followed by dynamic contrast-enhanced sequences. T1 weighted dynamic sequences were acquired previously as 15 mL gadolinium bolus injection (gadopentetic acid and dimeglumine salt, Magnevist; Schering, Berlin, Germany), administered with a 2 mL/sec flow, followed by a saline flush of 10 mL. MRI showed a contrast enhancement of the left nipple extending to subareola region, corresponding to the clinical lesion. The distribution pattern was segmental and linear, similar to the course of the ducts without a specific mass configuration (Figure 3). The enhancement was homogeneous, and time-signal intensity curve was slow and progressive (Figure 4). These results appeared suggestive for Paget's mammary disease, so the patient underwent an incisional biopsy of the left nipple, and a stereotactic biopsy in the area of microcalcifications was performed. Histological findings demonstrated the presence of a noninvasive Paget's disease with high grade of DCIS; therefore, a left mastectomy with sentinel lymph node (SLN) biopsy was performed. SLN was negative for metastasis.

## 3. Discussion

Paget's disease is a rare presentation of the breast cancer, accounting for 1%–4.3% of all breast female carcinomas, with a peak incidence between 50 and 60 years old [1, 2]. It is characterized by infiltration of neoplastic cells in the nipple epidermis, and it presents different histopathological patterns: it could be associated with DCIS and/or ductal invasive cancer; in only 8% of cases, Paget's disease occurs without any underlying neoplasia [4–6]. The pathogenesis of Paget's disease still remains controversial and supported by two different theories: intraepidermal transformation theory and the most reliable epidermotropic theory, that is associated with an underlying carcinoma [6–8]. Signs and symptoms usually occur in one breast and include itching, eczema, erythema of the nipple and areola, nipple erosion or ulceration, and retraction or bloody secretion; some patients have two or more symptoms at presentation, although often they are asymptomatic, and, occasionally, a palpable mass is detectable [1, 2, 9–11]. Mammary Paget's disease must be differentiated from other benign and/or malignant processes of nipple-areola complex such as atopic or contact dermatitis, chronic eczema, psoriasis, nipple duct adenoma, malignant melanoma, basal cell carcinoma, and Bowen's disease. In some cases, only biopsy and subsequent histological analysis allow a correct differential diagnosis [12].

In clinical suspicion, mammography can help to detect the underlying malignancy. Mammography findings include skin thickening, malignant calcification, or masses at the level of the nipple, architectural distortion, and nipple retraction. However, the literature reports that the mammography can be negative in 22%–50% of patients [2, 5, 13, 14]. As underlying carcinoma is common even in women with a benign mammogram and no palpable mass, the breast US and MRI may be useful in detecting the lesion. US can be considered a part of initial evaluation and helpful for increasing sensitivity of mammography. US findings include mass, ductal ectasia, flattening, asymmetry, and thickening of nipple and areola [15]. MRI is a diagnostic tool useful in the detection of clinically occult cancer with no mammographic and ultrasonographic signs of malignancy; in fact, MRI has a sensitivity of 95% compared to 70% of mammography in the detection of breast lesions [16]. MRI is useful not only to differentiate the normal nipple from the abnormal one but also to evaluate the extension of the tumor. The diagnosis is confirmed by biopsy. Mastectomy has been regarded for a long time as a standard therapy. Recently, conservative treatment, which involves the complete resection of the nipple-areola complex followed by radiation therapy, proved to be an alternative approach in patients with cancer confined to the central quadrant of the breast [17, 18]. Paget's disease associated with DCIS or invasive breast cancer treatment should include complete resection of the underlying disease with excision of the nipple-areola complex and radiation therapy of the remaining breast tissue [19].

## 4. Conclusion

In conclusion, MRI was a valid and accurate diagnostic tool for the diagnosis of pathology and has a better sensitivity and specificity in the evaluation of mammary Paget's disease related to mammography and US.
